# Supplementary material for: The Trithorax protein Ash1L promotes myoblast fusion by activating Cdon expression
Source: Nat Commun. 2018 Nov 28;9:5026. doi: 10.1038/s41467-018-07313-8 (PMC6262021; doi:10.1038/s41467-018-07313-8)
Supplement: Supplementary file 1 — Supplementary Information [file 41467_2018_7313_MOESM1_ESM.pdf]

SUPPLEMENTARY INFORMATION

The Trithorax protein Ash1L promotes myoblast fusion by activating Cdon expression

Ilaria Castiglioni, Roberta Caccia, Jose Manuel Garcia-Manteiga, Giulia Ferri, Giuseppina Caretti, Ivan Molineris, Kenichi Nishioka and Davide Gabellini*

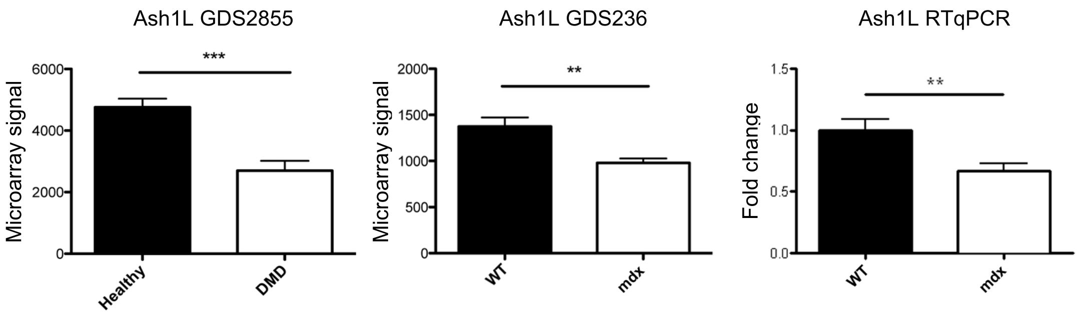

Supplementary Figure 1. Ash1L is significantly downregulated in Duchenne muscular dystrophy (DMD)
Ash1L expression profiles in DMD patients and mdx mice compared to control, from GEO database (GDS2855 and GDS236). RT-qPCR analysis of Ash1L expression in vastus lateralis of 2.5 months old mice: wild type (WT) vs mdx. Unpaired two-tailed t test. Confidence intervals 95%. n=6. **p≤0.01, ***p≤0.001.
Source data are provided as a Source Data file.

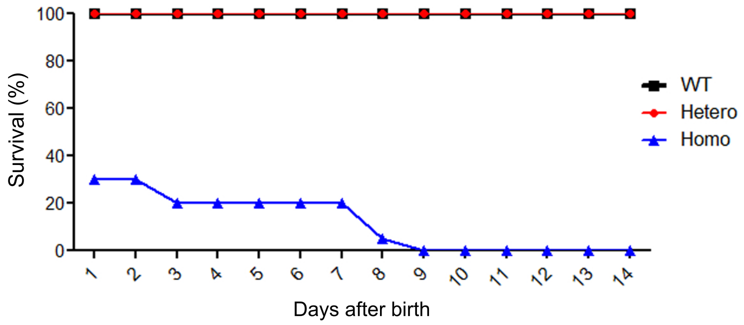

Supplementary Figure 2. Survival rate of WT, heterozygous and homozygous Ash1L GT mice.
Percentage of live animals of the indicated genotypes was measured every day after birth for 14 days. The majority of homozygous Ash1L GT mice don't survive past P1 and the rest of them are dead by P8.
Source data are provided as a Source Data file.

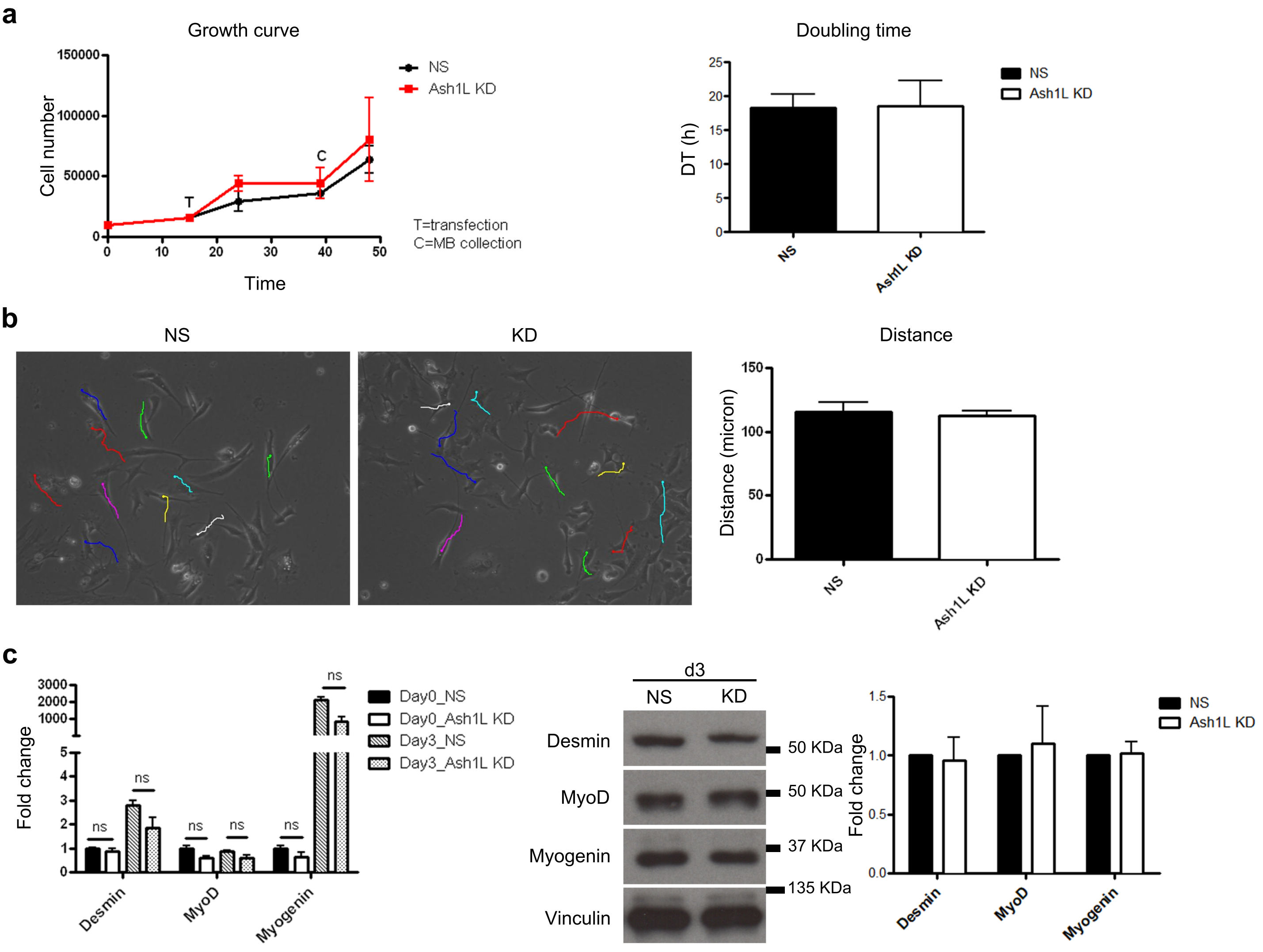

Supplementary Figure 3. Proliferation, migration and myogenic pathways of muscle cells are not affected by Ash1L depletion
a. Cell proliferation is not affected by Ash1L ablation. Growth curve and doubling times evaluation of C2C12 cells transfected with non-targeting (NS) or Ash1L siRNAs (Ash1L KD). Unpaired two-tailed t test. Confidence intervals 95%. n=3.
b. Migratory capability of C2C12 cells is not significantly altered by Ash1L ablation. Migration of muscle cells was quantified using time-lapse microscopy, as described in Material and Methods. The mean of three independent experiments was evaluated for a total of 99 cells tracked per conditions and expressed in distance (um) (right). Unpaired two-tailed t test. Confidence intervals 95%. n=3. On the left, a snapshot of two representative final time points, showing the migration tracks.
c. The myogenic markers Desmin, MyoD and Myogenin display no significant alteration upon Ash1L knockdown. RT-qPCR analysis on C2C12 cells transfected with non-targeting (NS) or Ash1L siRNAs (Ash1L KD), at day 0 and day3 of differentiation (left). Immunoblot, and densitometric analysis relative to Vinculin as housekeeping protein, at day 3 of differentiation (right). Paired two-tailed t test. Confidence intervals 95%. n=3.
Source data are provided as a Source Data file.

a

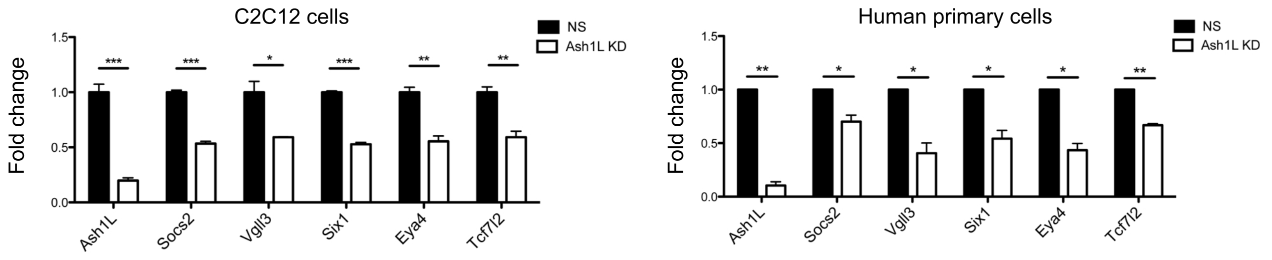

b

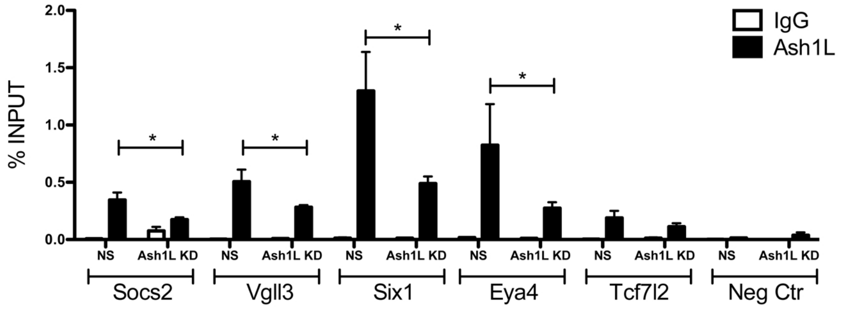

Supplementary Figure 4. Validation of the RNA-seq and ChIP-seq results

a. A representative list of direct Ash1L targets is significantly downregulated in Ash1L depleted muscle cells. RT-qPCR analysis on C2C12 (left) and human primary cells (right) transfected with non-targeting (NS) or Ash1L siRNAs (Ash1L KD) and collected at day 1 of differentiation. Unpaired two-tailed t test. Confidence intervals 95%. Results come from three independent experiments (C2C12 cells), and three healthy subjects (human samples).

b. ChIP-qPCR analysis on control (NS) vs Ash1L KD C2C12 cells. Ash1L enrichment on positive peak regions identified by ChIP-seq analysis and a negative control region. Ash1L enrichment on all Ash1L direct targets is decreased upon Ash1L ablation, indicating binding specificity. Paired one-tailed t test. Confidence intervals 95%. Results are from three independent experiments.

*p≤0.05, **p≤0.01, ***p≤0.001.

Source data are provided as a Source Data file.

a

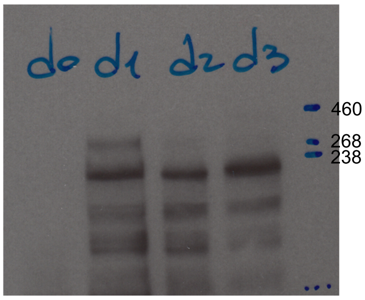

anti-Ash1L

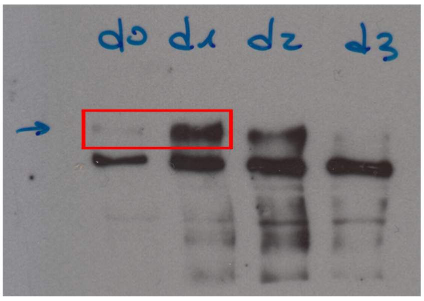

anti-Ash1L

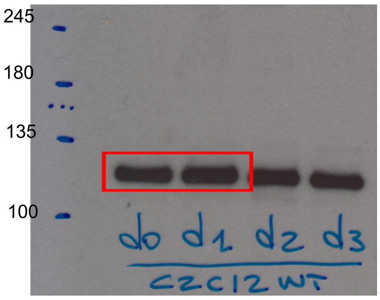

anti-Vinculin

Data shown in Fig. 1c

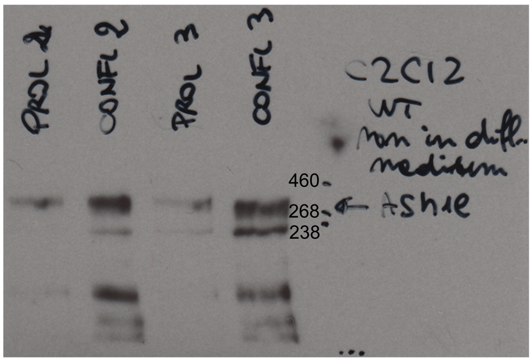

anti-Ash1L

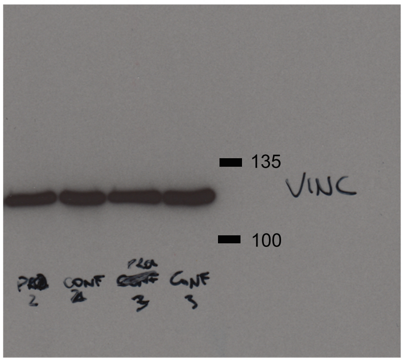

anti-Vinculin

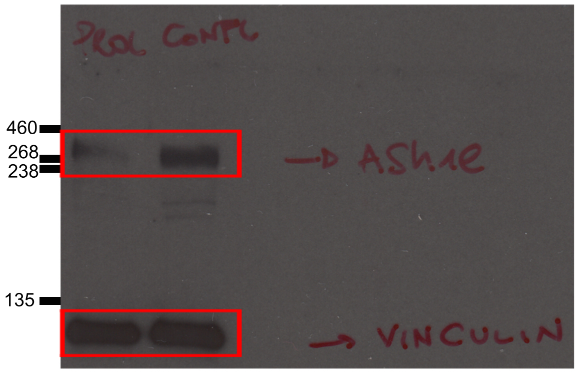

anti-Ash1L and anti-Vinculin

Data shown in Fig. 1d

b

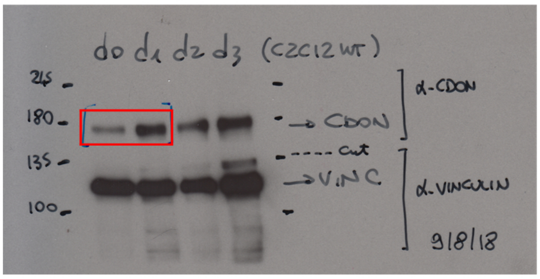

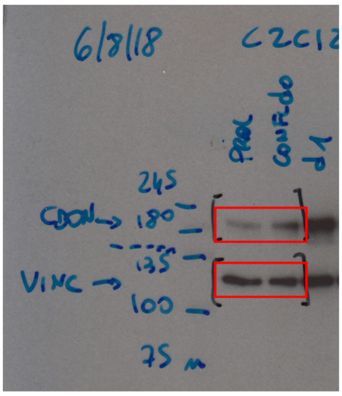

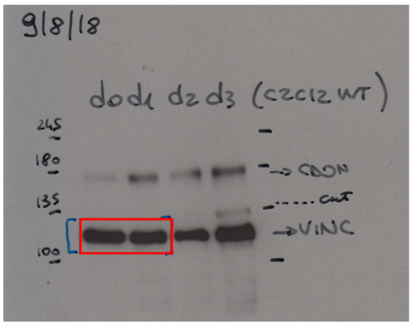

Data shown in Fig. 7c

c

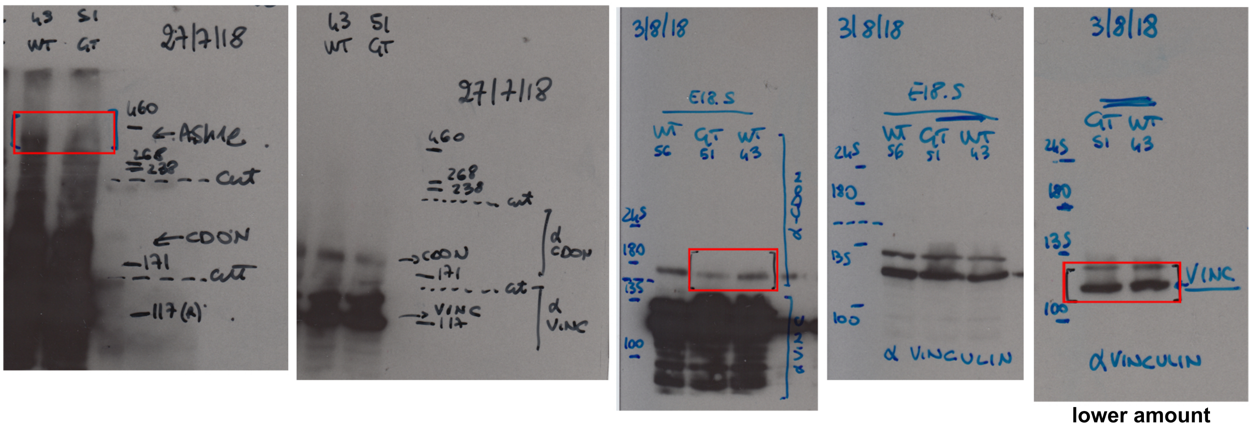

lower amount

same gel

same gel

d

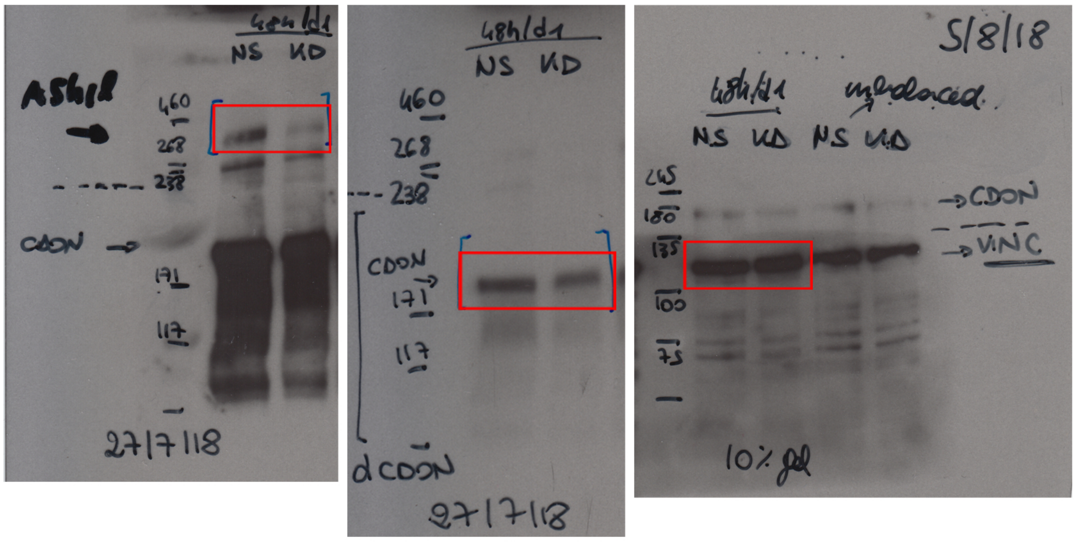

same gel

Data shown in Fig. 7e

e

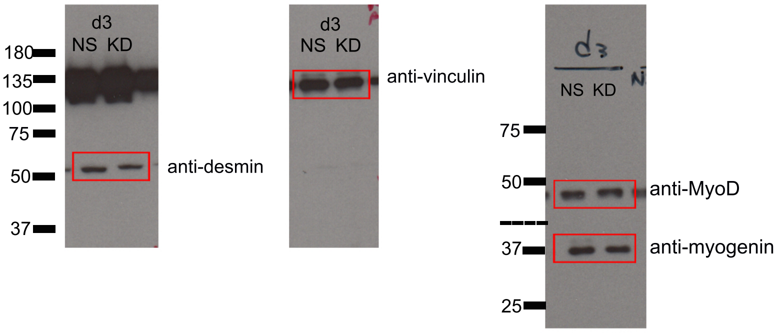

Data shown in Suplementary Figure 3c

Suplementary figure 5. Uncropped represantive immunoblots.
a. Protein extract from C2C12 cells. Staining with primary antibodies anti-Ash1L and anti-Vinculin.
b. Protein extract from C2C12 cells. Staining with primary antibodies anti-Cdon and anti-Vinculin.
c. Protein extract from WT and Ash1L GT muscle tissue. Staining with primary antibodies anti-Ash1L, anti-Cdon and anti-Vinculin.
d. Protein extract from C2C12 cells. Staining with primary antibodies anti-Ash1L, anti-Cdon and anti-Vinculin.
e. Protein extract from C2C12 cells at day 3 of diferentiation. Staining with primary antibodies anti-desmin, anti-MyoD, anti-myogenin and anti-Vinculin.

a

Gene	Species	Application	Sequence
Gapdh	mouse/human	RT-qPCR	F 5'-TCAAGAAGGTGGTGAAGCAGG-3'
			R 5'-ACCAGGAAATGAGCTTGACAAA-3'
Ash1L	mouse	RT-qPCR	F 5'-GCCCTTCAAAATGGGACTCT-3'
			R 5'-TCGACTGCCAACAAGAGATG-3'
Cdon	mouse	RT-qPCR	F 5'-CGGTTGGACAGAAACACAGA-3'
			R 5'-CGAAATCACCCAGGGCTG-3'
MyoD	mouse	RT-qPCR	F 5'-AGTGAATGAGGCCTTCGAGA-3'
			R 5'-CTGGGTTCCCTGTTCTGTGT-3'
Myogenin	mouse	RT-qPCR	F 5'-ACTCCCTTACGTCCATCGTG-3'
			R 5'-CAGGACAGCCCCACTTAAAA-3'
Desmin	mouse	RT-qPCR	F 5'-ATGAAGAGGAGATCCGTGAG-3'
			R 5'-GGTATTCCATCATCTCCTGCT-3'
MyHC4-2b	mouse	RT-qPCR	F 5'-CACCTGGACGATGCTCTCAGA-3'
			R 5'-GCTCTTGCTCGGCCACTCT-3'
M-Cadherin	mouse	RT-qPCR	F 5'-CCTGATGGGCAGTTCAAGATC-3'
			R 5'-CACGGACAGCACACCTTCAT-3'
Myomaker	mouse	RT-qPCR	F 5'-ATCGCTACCAAGAGGCGTT-3'
			R 5'-CACAGCACAGACAAACCAGG-3'
Socs2	mouse	RT-qPCR	F 5'-GGACGTGTTGACTCATCTCC-3'
			R 5'-ACCATCCTGTTTGACTGAGC-3'
Vgll3	mouse	RT-qPCR	F 5'-GGCTTCGATACAGGTCTTCA-3'
			R 5'-CATTTCCTGTGTCCCATTTC-3'
Six1	mouse	RT-qPCR	F 5'-CCAGGTCAGCAACTGGTTTA-3'
			R 5'-CTCCTCTTCTGAGCTGGACA -3'
Eya4	mouse	RT-qPCR	F 5'-AGTACTCTGGGATGCAGCAG-3'
			R 5'-AGTAGACGATGGTGCAAAGC-3'
Tcf7l2	mouse	RT-qPCR	F 5'-GGAGGAGAAGAACTCGGAAA-3'
			R 5'-CGGGATTTATCTCGGAAACT-3'
Ash1L	human	RT-qPCR	F 5'-GAAGACCTTTTCCGGGTAGG-3'
			R 5'-GTAGTGGGGACCTGCTGTGT-3'
Cdon	human	RT-qPCR	F 5'-AGTGAAACGTGTTCCTGGAG-3'
			R 5'-AACCATCAGAATGAGGACCA-3'
Socs2	human	RT-qPCR	F 5'-GCACTGACTTCAAGGAAGGA-3'
			R 5'-CTTCCCCAGTACCATCCTGT-3'
Vgll3	human	RT-qPCR	F 5'-CTCGTGTGTCCTTTTCACT-3'
			R 5'-CTGAGCTGTCTCGCCATAG-3'
Six1	human	RT-qPCR	F 5'-ATTCTCACCTCCCCAAAGTC-3'
			R 5'-GAGAGTCTTGGAGCTGATGC-3'
Eya4	human	RT-qPCR	F 5'-GTGGCAGGAGAAGTGAGAAA-3'
			R 5'-GGTTTCACTGCTGTTCAAGG-3'
Tcf7l2	human	RT-qPCR	F 5'-CCCCGTATTACCCACTATCG-3'
			R 5'-GAACCTGGACATGGAAGCAT-3'
Cdon Positive peak region	mouse	ChIP-qPCR	F 5'-GCTTTAGCACTGAAGCGAAC-3'
			R 5'-AAGCCTGACTTTGCTCTCAA-3'
Cdon Negative region	mouse	ChIP-qPCR	F 5'-AGTGAGGGTCGTGCTTTGTA-3'
			R 5'-GACTGGTGGTGTAATGCTGC-3'
Socs2 Positive peak region	mouse	ChIP-qPCR	F 5'-CAGCTGCGATTCATACAGTC-3'
			R 5'-GGGCTCATTTTGGAAATGTT-3'
Vgll3 Positive peak region	mouse	ChIP-qPCR	F 5'-GCGTGAAAGATGCTTTAGGA-3'
			R 5'-AGACAGATGCCTTTCGTGAG-3'
Six1 Positive peak region	mouse	ChIP-qPCR	F 5'-AGGGGAGAAGGGAGACTTTT-3'
			R 5'-TGCTGGGTGTCCTATCAGAG-3'
Eya4 Positive peak region	mouse	ChIP-qPCR	F 5'-AGGCTCAGTCAAAACCTCCT-3'
			R 5'-CGCTTGTAGAGCTTGGTCAT-3'
Tcf7l2 Positive peak region	mouse	ChIP-qPCR	F 5'-GGCCTTTTGAGTGGACTTTT-3'
			R 5'-CCAAACCTCCTACACCTCCT-3'
Negative control region	mouse	ChIP-qPCR	F 5'-TCTGCCCATTTTCATACACA-3'
			R 5'-TGTCACAGTTCGTGATGGTC-3'

b

**Western Blot Antibodies**
Rabbit anti-Ash1L (#A301-749A; Bethyl laboratories)
Mouse anti-Vinculin (#V9131; Sigma-Aldrich)
Mouse anti-CDO (#AF2429; R&D Systems)
Mouse anti-MyoD (#M3512; Dako)
Mouse anti-Myogenin F5D (Hybridoma Bank)
Rabbit anti-Desmin (#4024; Cell Signalling)
Peroxidase-AffiniPure Donkey Anti-Rabbit IgG (H+L) (#JI711035152; Jackson ImmunoResearch)
Peroxidase-AffiniPure Donkey Anti-Mouse IgG (H+L) (#JI715035150; Jackson ImmunoResearch)
Peroxidase-AffiniPure Donkey Anti-Goat IgG (H+L) (#705-035-003; Jackson ImmunoResearch)

**ChIP Antibodies**
Rabbit IgG (#011-000-003; Jackson Immunoresearch)
Rabbit anti-Ash1L (#A301-749A; Bethyl laboratories)
Rabbit anti-H3 (#Ab1791; Abcam)
Rabbit anti-dimethyl-Histone H3 (Lys 36) (#07-369; Millipore)

**IF Antobodies**
Primary antobodies
Rabbit anti-Ash1L (#A301-749A; Bethyl laboratories)
Rabbit anti-Laminin (#L9393; Sigma)
Secondary antobodies
Alexa Fluor 488 goat anti-rabbit (#A-11034, Molecular Probes)

Suplementary Table 1
a. Sequences of the primers used in RT-qPCR and in ChIP-qPCR
b. List of antibodies used in this study
