# Supplementary material for: The Trithorax protein Ash1L promotes myoblast fusion by activating Cdon expression
Source: Nat Commun. 2018 Nov 28;9:5026. doi: 10.1038/s41467-018-07313-8 (PMC6262021; doi:10.1038/s41467-018-07313-8)
Supplement: Supplementary file 9 — Source Data Fig. 1 [file 41467_2018_7313_MOESM9_ESM.docx]

**Title:** Supplementary Data 1.
**Description:** List of differentially expressed genes in myoblasts knockdown for Ash1L compared to control knockdown.

**Title:** Supplementary Data 2.
**Description:** Lis of Ash1L ChIP-Seq peaks annotated based on their position relative to genes.

**Title:** Supplementary Data 3.
 **Description:** List of direct Ash1L targets differentially expressed in myoblasts knockdown for Ash1L compared to control knockdown.

**Title:** Supplementary Data 4.
**Description:** Differential gene expression of Ash1L targets during C2C12 differentiation. Supplementary Data 5. ChIP-Seq Enrichment Analysis by Enrichr for direct Ash1L target genes.

**Title: Source data file**

**Description**: The source data underlying Figures 1a, 1b, 1c, 1d, 2a, 2b, 3a, 3b, 3c, 7a, 7b, 7c, 7d, 7e, 7f, 8b, 8c and Supplementary Figures 1, 2, 3a, 3b, 3c, 4a and 4b are provided as a Source Data file.
